# Single-port laparoscopic percutaneous extraperitoneal closure for inguinal hernias repair in girls: using an epidural needle assisted by a towel forceps

**DOI:** 10.1186/s12893-020-00800-0

**Published:** 2020-06-19

**Authors:** Yuanhong Xiao

**Affiliations:** Department of Pediatric Surgery, the First Medical Center, Chinese PLA General Hospital, Fuxing Road 28th, Haidian District, Beijing, 100853 China

**Keywords:** Child, Girls, Inguinal hernia, Laparoscopy, Minimal invasive surgery

## Abstract

**Background:**

The concept of single-port laparoscopic percutaneous extraperitoneal closure for the treatment of inguinal hernias repair in children has been practising recent years. The applicable instruments and skills are still evolving. In this study, we used an epidural needle assisted by a towel forceps to practise this minimal invasive method for inguinal hernias repair in girls. Its safety and effectiveness were studied.

**Methods:**

From July 2008 to January 2020, thirty-five girls diagnosed of indirect inguinal hernias were studied retrospectively. From April 2017 to January 2020, the author was free to propose open or laparoscopic repair for the patients. The open group included twenty-four girls and the laparoscopic group included eleven. The data of the patients age, constituent ratios of sliding and bilateral hernias, operating time, postoperative time in hospital, follow-up time, conversion, postoperative complications were assessed.

**Results:**

There were no statistically significant difference between the laparoscopic group and open group for the following items: age, operating time, postoperative time in hospital, the constituent ratios of sliding hernia and bilateral hernias (*P* > 0.05). The follow-up time of the open group was longer than that of the laparoscopic group (*P* = 0.0004). One laparoscopic case was converted to open surgery. After 12 cases of laparoscopic practice, coordination of the hand and eye movements established well. There were no postoperative complications for all the patients.

**Conclusion:**

Our preliminary experience suggested that it is safe and convenient for inguinal hernias repair in girls by the single-port laparoscopic percutaneous extraperitoneal closure method using an epidural needle assisted by a towel forceps.

## Background

The concept of single-port laparoscopic percutaneous extraperitoneal closure for the treatment of inguinal hernias repair in children has been practised recent years [1–11]. The applicable instruments and skills are still evolving and being probed, such as modified Kirschner pin, epidural needle, optical forceps assisting, two-hooked cannula device and two-hooked core needle apparauts et al [1, 2, 7, 8, 10] In this study, we used an epidural needle assisted by a towel forceps to practise this minimal invasive method for inguinal hernias repair in girls. Its safety and effectiveness were studied.

## Methods

### Population and data collection

From July 2008 to January 2020, thirty-five girls diagnosed of indirect inguinal hernias were studied retrospectively. From April 2017 to January 2020, the author was free to propose open or laparoscopic repair of the patient. The laparoscopic group included 11 girls with 16 affected sides including one sliding hernia. The open group included 24 girls with 29 affected sides including 4 sliding hernias (Table 1). The data of age, constituent ratios of sliding hernia and bilateral hernias, operating time, postoperative time in hospital, follow-up time, conversion to open surgery, postoperative complications were assessed (Tables 1 and 2). All the parents signed the informed contents for open or laparoscopic surgery.

**Table 1:** Patient characteristics

Items	Open group	Laparoscopic group	*P* value
Age(y)	5.633 ± 4.538	5.952 ± 3.402	0.8366
	(0.11–15)	(1.08–13)	
Cases	24	11	
Sides	29	16	
Left	11	3	
Right	8	3	
Bilateral	5	5	0.4793
Slidding	4	1	0.7831

**Table 2:** Patient outcomes

Items	Open group	Laparoscopic group	*P* value
Operating time (min)	45.958 ± 17.289	56.272 ± 20.533	0.1318
	(28–85)	(22–83)	
Postoperative time (d)	3.083 ± 1.442	2.818 ± 0.405	0.5561
	(1–7)	(2–3)	
Follow-up time (m)	71.313 ± 46.781	14.455 ± 12.809	0.0004
	(0.5–139)	(1–34)	

### Surgical processes

**Fig. 1:**
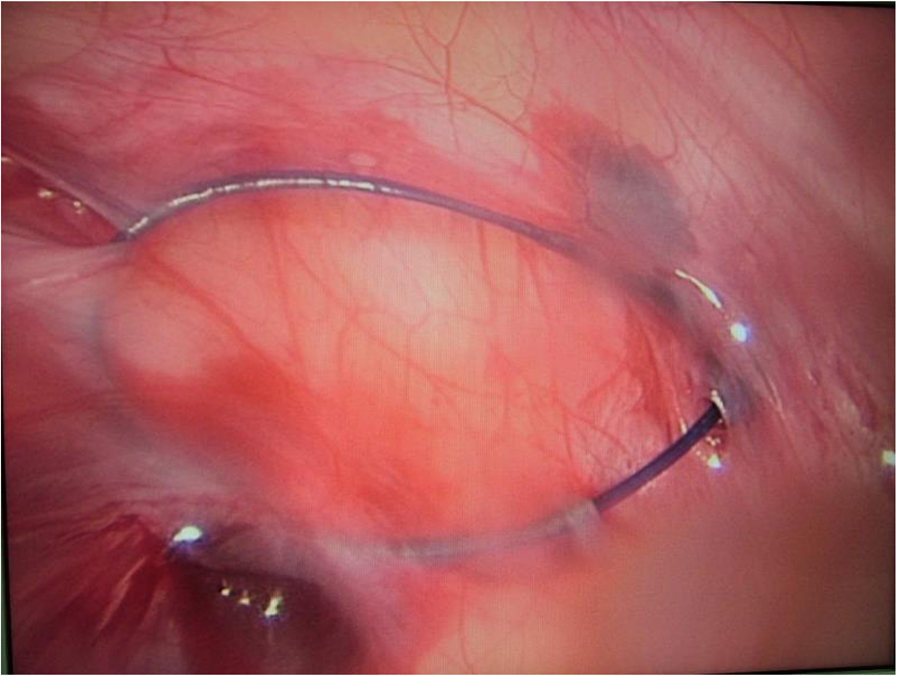
Thread circling of the internal ring. The image shows a Prolene thread has circled the internal ring preperitoneally

**Fig. 2:**
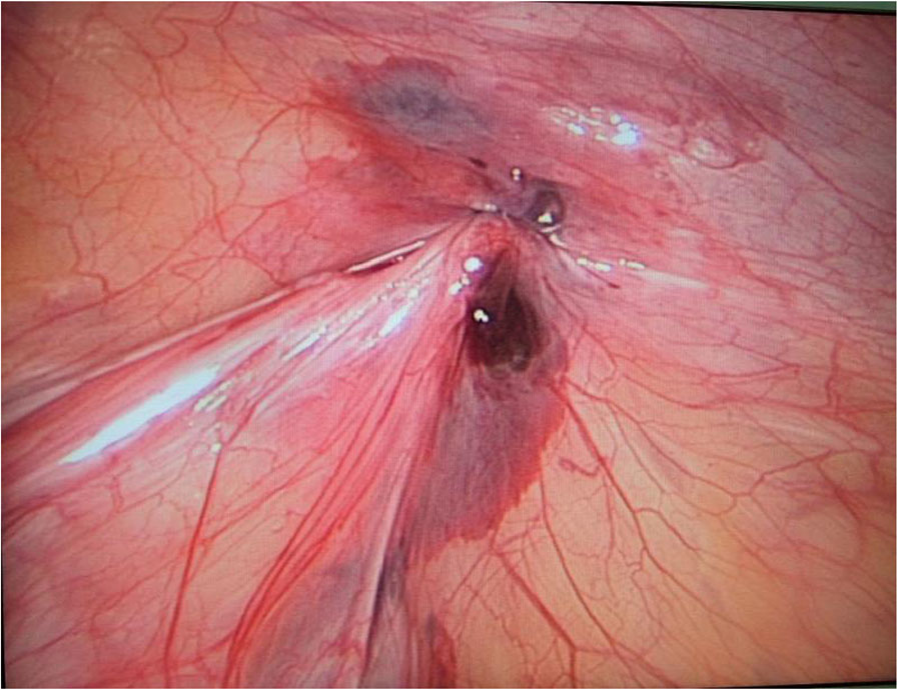
Closure of the internal ring. The image shows the Prolene thread has been tied outside of the body to close the internal ring

**Fig. 3:**
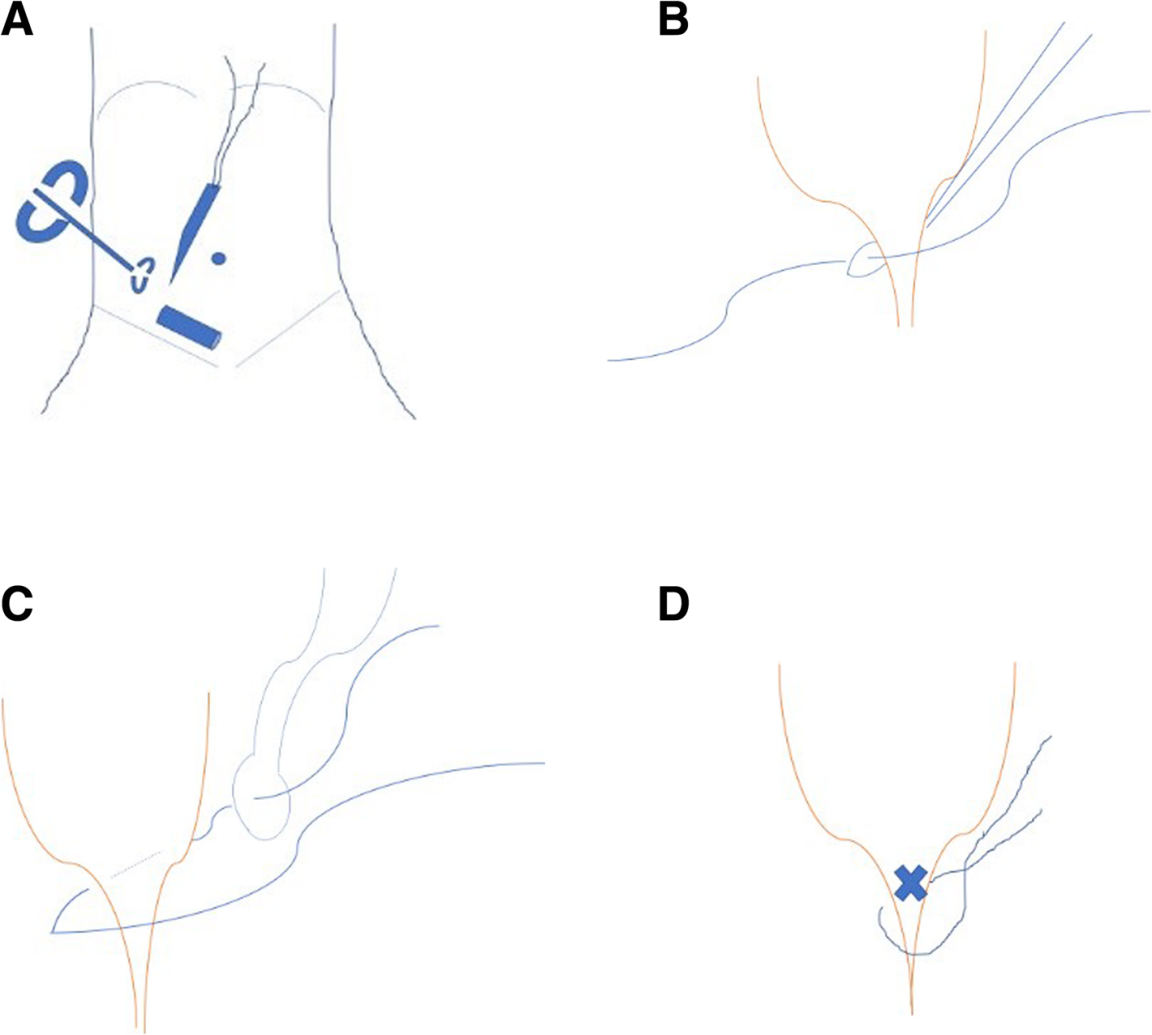
Schematic diagram illustrating the principle of the technique. **a**. Puncturing instrument with a towel assistance. The image shows the epidural needle with a Prolene loop inside of the barrel is puncturing preperitoneally, with a towel forceps stretching of the groin skin as an assistance. This maneuver inclines the operator to establish an eye-hand coordination. **b**. Circling thread capture by the thread loop. The image shows the circling thread has been trapped by the Prolene thread loop. **c**. Thread circling completion by the thread loop. The image shows the single Prolene thread has been trapped and pulled out of the body by the Prolene thread loop. This single Prolene thread has circled the internal ring completely. **d**. The internal ring closure. The image shows the single Prolene thread is being knot-tied to close the internal ring outside of the body at the groin site

Patient lay in the supine position under general anesthesia. For open repair, classical technique was used: incising the skin, Scarpa’s fascia, external oblique aponeurosis; dissecting and seperating the sac from the spermatic cord; high ligating the sac neck at the level of the internal ring. For laparoscopic operation, the surgeon stood at the left side of the patient regardless of the affected side of the hernia. The screen was placed at the patient feet side. One cm arc incision along the lower edge of the umbilicus was made. After blunt dissection of the subcutaneous tissue with a mosquito clamp, a pneumoperitoneum needle was inserted through the incision to the abdominal cavity. Then, carbon dioxide was poured into the cavity with a pressure of 8–12 mmHg. When the abdominal cavity was distended, the pneumoperitoneum needle was withdrawn and lamp trocar (10 mm, 30° optics) was inserted through the umbilical incision. Peritoneal cavity was inspected first to preclude accidental injuries to the abdominal wall and peritoneal viscera. Re-confirmation of the affected side was made through a direct vision image. The image of the cavity was captured by the camera lens and appeared on the screen. In our group, through a careful groin examination and an ultrasound inspection, we found no diagnostic neglection of the bilateral hernias preoperatively. A tiny incision of 2 mm was made on the affected groin side. The incision was accurately positioned just above the level of the internal ring with the help of the camera lens. An epidural needle with a 3–0 Prolene loop inside the barrel was used. The 3–0 Prolene loop was inserted into the barrel by the operator beforehand. The epidural needle was characteristic with the followings: 1.8 mm × 80 mm, TUORen, Medical Instrument Group Corporation, MengGang Reed Garden Industrial District, ChangHeng County, Henan, China. The operator held the needle with one hand; a towel forceps stretching the groin skin with the other hand. The needle was pierced to the preperitoneal space through the groin tiny incision. Under the view of the laparoscopic lens, with the streching help by the towel forceps, the needle passed through a semicircle of the internal ring preperitoneally. Then, the needle tip pierced the peritoneum into the peritoneal cavity. The Prolene loop was pushed into the peritoneal cavity through the barrel of the needle. Maintaining the loop in the cavity, the needle was withdrawn carefully out of the body until the two ends of the 3–0 Prolene seperated from the needle. The needle was introduced again from the same tiny incision of the groin skin and pierced into the site of the preperitoneal space, nearly the same site with the first time. Then, the needle was passed through the other semicircle of the internal ring preperitoneally. When the needle tip reached the previously pierced point of the peritoneum where the loop was still inside, it was managed to pierce the peritoneum and pass through the loop. Maintaining the tip inside the loop of 3–0 Prolene, one 2–0 Prolene was inserted into the barrel and continuously into the loop of the 3–0 Prolene for a suitable length. Maintaining the 2–0 Prolene trapped by the 3–0 Prolene loop, the needle was withdrawn once more outside of the body until the other end of the 2–0 Prolene seperating from the needle. The operator took the two ends of the loop and pulled them outwards. When the loop was pulled outside of the body, one end of the trapped 2–0 Prolene in the loop will be brought out of the preperitoneal cavity simultaneously. At this moment, two ends of the 2–0 Prolene were all outside of the body at the tiny incision site. A circle had been formed preperitoneally at the level of the internal ring (Fig. 1). Knot tying of the 2–0 Prolene was made outside of the body and buried under the groin skin. Then the internal ring closing preperitoneally was completed (Fig. 2). A schematic diagram demonstrating the main steps and principle of the technique was shown in Fig. 3. Commonly, the round ligament will be included into the cerclage. The umbilical incision and the puncturing tiny incision were closed with absorbable suture by subcutaneous suture and intradermal suture. The incision was covered with a waterproof dressing. During the whole procedure, extreme care was taken not to damage the inferior epigastric or femoral vessels.

### Data analysis

Data analysis was performed using CHISS (Chinese High Intellectualized Statistical Software) software version 2004. Student T Test was used to compare the distribution of quantitative variables between groups (P65–68, Medical Statistics and CHISS Application) [12]. Pearson Chi-square Test was used to compare the distribution of the qualitative variables between groups (P110–118, Medical Statistics and CHISS Application) [12]. Statistical significance was defined as *P* value< 0.05.

## Results

There were no statistically significant differences of patients age, operating time, postoperative time of staying in hospital between the open group and laparoscopic one (*P* = 0.8366, 0.1318, 0.5561, respectively). There was no statistical constituent ratios difference of the bilateral hernias and sliding hernias between the two groups (*P* = 0.4793, 0.7831, respectively). The follow-up time for open group was significantly longer than the laparoscopic group (*P* = 0.0004) (Table 2). There were no postoperative complications for all the patients. One girl in the laparoscopic group was converted to open surgery for a sliding hernia. After 12 cases of single-port laparoscopic practice assisted by a towel forceps stretching on the groin skin, coordination of the hand and eye movements began to form well.

## Discussion

The general circumstances of the open group and laparoscopic one were comparable for there were no statistically significant differences on age, constituent ratios of sliding and bilateral hernias. There was no statistically significant difference of the operating time and post-operative time in hospital. During the follow-up periods, there was no complications for all the patients although the laparoscopic group had been followed-up for a shorter time. From the results, we can propose that minimal invasive concept of single-port laparoscopic percutaneous extraperitoneal closure for inguinal hernias repair in girls was acceptable and practicable, which has been suggested by many recent studies [1–11]. The application of an epidural needle assisted by the towel forceps was convenient, effective and safe. Because of the smaller abdominal cavity in children, especially infants, it is important to separate the cannulas as widely as possible to provide adequate working space for an efficient performance of the operation. Placement of the ports too closely hinders the operation and most likely diminishes the advantages of this approach [13]. The novel method of single-port laparoscopic technique obviously avoids of this circumstance.

Infants and young children have pliable abdominal walls. Therefore, it is necessary to advance the instrument cautiously as it penetrates the peritoneum to prevent injury to the underlying viscera and intestine [13]. The epidural needle employed was disposable with a slightly bending dull tip like a shovel which endowed it a suitable sharpness for puncturing conveniently. There was no instrument damage in this study. The barrel of it was wide enough for two Prolene threads to pass through without obstruction. Prolene thread was easy to maintain a loop in the peritoneal cavity due to its appropriate solidity.

Proprioceptive instrument positioning, eye-hand coordination, spatial visualization, manual dexterity and rapid mental processing were all important in the acquisition of laparoscopic skills [14–20]. In this study, we applied a traction of the groin skin with a towel forceps when puncturing the epidural needle to form a bilateral hands manipulation in the operative field. This method will help the operator to form a position perception of the needle while it was passing through the preperitoneal space. For a beginner, with one hand manipulation of the needle, it was difficult to establishe the position sense of the instrument and resulted in a chaotic needle insertion, even a conversion to an open operation. In addition, with gazing the needle piercing route along the preperitoneum adjacent to the internal ring continuously, the surgeon developed effective eye-hand coordination which accelerated the procedure and enhanced its safety. For this study, the author discovered the eye-hand coordination establishment when 12 cases performance had been accomplished. More data should be accumulated to study the eye-hand coordination for this method in the future.

Another special concern in children is excessive abdominal insufflation. There was marked acidemia, hypoxia, and increased exhaled CO_2_ with higher insufflation pressures. Some authors recommended using insufflation pressure of less than 15 mmHg. Many pediatric surgeons, however, continue to use 15 mmHg as the maximum inflating pressure without apparent adverse clinical sequelae [13]. In this study, 8–12 mmHg pressure was used without side effects.

## Conclusions

Our preliminary experience suggested that it is safe and convenient for inguinal hernias repair in girls by the single-port laparoscopic percutaneous extraperitoneal closure method using an epidural needle assisted by a towel forceps.

## Data Availability

All data is contained within the manuscript and its additional files. The datasets used and analysed during the current study available from the corresponding author on reasonable request.

## Abbreviations

CHISS: Chinese High Intellectualized Statistical Software

## References

[1] Yonggang H, Changfu Q, Ping W, Fangjie Z, Hao W, Zicheng G (2019). Single-port laparoscopic percutaneous extraperitoneal closure of inguinal hernia using "two-hooked" core needle apparatus in children. Hernia..

[2] Li S, Liu X, Wong KKY, Liu L, Li Y (2018). Single-port laparoscopic herniorrhaphy using a two-hooked cannula device with hydrodissection. J Pediatr Surg.

[3] Xu L, Li CQ, Chen XD, Qiu MJ, Jiang JH, Yao C (2017). Laparoscopic percutaneous extraperitoneal closure does not affect vas deferens orientation or testicular volume and perfusion. Zhonghua Nan Ke Xue.

[4] Korkmaz M, Güvenç BH (2018). Comparison of single-port percutaneous Extraperitoneal repair and three-port mini-laparoscopic repair for pediatric inguinal hernia. J Laparoendosc Adv Surg Tech A.

[5] Li C, Xu L, Peng Y, Liang X, Lin W (2016). Effects of single-port laparoscopic percutaneous extraperitoneal closure on the orientation of the vas deferens and testicular perfusion and volume: Experience from a single center. J Pediatr Urol.

[6] Timberlake MD, Sukhu HKW, Rasmussen S, Corbett ST (2015). Laparoscopic percutaneous inguinal hernia repair in children: review of technique and comparison with open surgery. J Pediatr Urol.

[7] Yilmaz E, Afsarlar CE, Senel E, Cavusoglu YH, Karaman I, Karaman A (2015). A novel technique for laparoscopic inguinal hernia repair in children: single-port laparoscopic percutaneous extraperitoneal closure assisted by an optical forceps. Pediatr Surg Int.

[8] Li S, Li M, Wong KK, Liu L, Tam PK (2014). Laparoscopically assisted simple suturing obliteration (LASSO) of the internal ring using an epidural needle: a handy single-port laparoscopic herniorrhaphy in children. J Pediatr Surg.

[9] McClain L, Streck C, Lesher A, Cina R, Hebra A (2015). Laparoscopic needle-assisted inguinal hernia repair in 495 children. Surg Endosc.

[10] Liu W, Wu R, Du G (2014). Single-port laparoscopic extraperitoneal repair of pediatric inguinal hernias and hydroceles by using modified Kirschner pin: a novel technique. Hernia..

[11] Chang YT, Lin JY, Lee JY, Tsai CJ, Chiu WC, Chiu CS (2012). Comparative mid-term results between inguinal herniotomy and single-port laparoscopic herniorrhaphy for pediatric inguinal hernia. Surg Laparosc Endosc Percutan Tech.

[12] Li G, Wang HW, Tong XY, Wang HY, Guo XH (2006). Matched Pairs Design Comparison. Medical Statistics and CHISS Application.

[13] Holcomb GW, Oldham KT, Colombani PM, Foglia RP (1997). Clinical principles of abdominal surgery. Surgery of infants and children: scientific principles and practice.

[14] Yoshida S, Fukuyo T, Saito K, Kihara K, Fujii Y (2017). Real-time three-dimensional image angle rectification to improve hand-eye coordination in single-port laparoendoscopic surgery. Int J Urol.

[15] Sindram D, IH MK, Martinie JB, Iannitti DA (2010). Novel 3-D laparoscopic magnetic ultrasound image guidance for lesion targeting. HPB-(Oxford).

[16] Honeck P, Wendt Nordahl G, Rassweiler J, Knoll T (2012). Three-dimensional laparoscopic imaging improves surgical performance on standardized ex-vivo laparoscopic tasks. J Endourol.

[17] Lusch A, Bucur PL, Menhadji AD, Okhunov Z, Liss MA, Perez-Lanzac A (2014). Evaluation of the impact of three-dimensional vision on laparoscopic performance. J Endourol.

[18] Mashiach R, Mezhybovsky V, Nevler A, Gutman M, Ziv A, Khaikin M (2014). Three-dimensional imaging improves surgical skill performance in a laparoscopic test model for both experienced and novice laparoscopic surgeons. Surg Endosc.

[19] Middleton KK, Hamilton T, Tsai PC, Middleton DB, Falcone JL, Hamad G (2013). Improved nondominant hand performance on a laparoscopic virtual reality simulator after playing the Nintendo Wii. Surg Endosc.

[20] Xu M, Shuyi W, Shasha Y (2017). Study on method of laparoscopic training based on eye gaze tracking techniques. J Biomed Eng.

